# Transcriptome signatures preceding the induction of anti-stalk antibodies elicited after universal influenza vaccination

**DOI:** 10.1038/s41541-022-00583-w

**Published:** 2022-12-10

**Authors:** Teresa Aydillo, Ana S. Gonzalez-Reiche, Daniel Stadlbauer, Mary Anne Amper, Venugopalan D. Nair, Chiara Mariottini, Stuart C. Sealfon, Harm van Bakel, Peter Palese, Florian Krammer, Adolfo García-Sastre

**Affiliations:** 1 Department of Microbiology, Icahn School of Medicine at Mount Sinai, New York, NY USA; 2 Global Health and Emerging Pathogens Institute, Icahn School of Medicine at Mount Sinai, New York, NY USA; 3 Department of Genetics and Genomics Sciences, Icahn School of Medicine at Mount Sinai, New York, NY USA; 4 Department of Neurology, Icahn School of Medicine at Mount Sinai, New York, NY USA; 5 Department of Medicine, Icahn School of Medicine at Mount Sinai, New York, NY USA; 6 Department of Pathology, Molecular and Cell Based Medicine, Icahn School of Medicine at Mount Sinai, New York, NY USA; 7 Department of Medicine, Division of Infectious Diseases, Icahn School of Medicine at Mount Sinai, New York, NY USA; 8 The Tisch Cancer Institute, Icahn School of Medicine at Mount Sinai, New York, NY USA; 9 Present Address: Moderna, Cambridge, MA USA

**Keywords:** Protein vaccines, Drug development

## Abstract

A phase 1 clinical trial to test the immunogenicity of a chimeric group 1 HA (cHA) universal influenza virus vaccine targeting the conserved stalk domain of the hemagglutinin of influenza viruses was carried out. Vaccination with adjuvanted-inactivated vaccines induced high anti-stalk antibody titers. We sought to identify gene expression signatures that correlate with such induction. Messenger-RNA sequencing in whole blood was performed on the peripheral blood of 53 vaccinees. We generated longitudinal data on the peripheral blood of 53 volunteers, at early (days 3 and 7) and late (28 days) time points after priming and boosting with cHAs. Differentially expressed gene analysis showed no differences between placebo and live-attenuated vaccine groups. However, an upregulation of genes involved in innate immune responses and type I interferon signaling was found at day 3 after vaccination with inactivated adjuvanted formulations. Cell type deconvolution analysis revealed a significant enrichment for monocyte markers and different subsets of dendritic cells as mediators for optimal B cell responses and significant increase of anti-stalk antibodies in sera. A significant upregulation of immunoglobulin-related genes was only observed after administration of adjuvanted vaccines (either as primer or booster) with specific induction of anti-stalk *IGVH1-69*. This approach informed of specific immune signatures that correlate with robust anti-stalk antibody responses, while also helping to understand the regulation of gene expression induced by cHA proteins under different vaccine regimens.

## Introduction

How influenza virus vaccines can be designed to induce strong broadly cross-reactive antibody responses is still debated and intensely studied^1^. Most commonly used influenza virus vaccines contain four different vaccine strains: two influenza A virus components belonging to the H1N1 and H3N2 subtypes; and two influenza B virus components derived from the B/Victoria/2/87-like and B/Yamagata/16/88-like lineages. The vaccine strains are selected based on worldwide surveillance and prediction methods to determine the most likely circulating antigenic variants of the upcoming influenza season. However, circulating influenza virus strains are continuously evolving and surveillance efforts are not equally effective around the world and may result in mismatches between circulating strains and vaccine strains leading to reduced efficacy^2^. Influenza virus vaccines are updated annually due to the rapid accumulation of mutations in the haemagglutinin (HA)—and to a lesser extent—in the neuraminidase (NA) genes of the viruses. This phenomenon, known as antigenic drift, originates from the ability of influenza viruses to escape pre-existing immunity in humans. A second mechanism to evade immune recognition is known as antigenic shift based on the acquisition of genes coding for novel surface proteins. Antigenic shift is responsible for the emergence of pandemic influenza virus strains. While the current licensed seasonal influenza virus vaccines are still the best preventive measure against influenza virus infection^3,4^, they are strain-specific and have a narrow coverage. We need improved strategies that can provide broad and long-lasting protection against multiple influenza virus strains, including pandemic influenza viruses. In addition, the development of improved next-generation universal influenza virus vaccines would also prevent seasonal vaccine failure due to mismatches between the predicted strains and the circulating influenza viruses.

We have developed chimeric HA (cHA) vaccination strategies targeting conserved regions of the HA surface protein, particularly the stalk domain^5–7^. In contrast to the head of the HA, the stalk domain is relatively conserved^8,9^. However, immune responses are largely targeted against the head, which is immunodominant and rather permissive to mutations^2,10^. To overcome this, the cHAs strategy exposes the immune system sequentially to constructs that share the same stalk domain (group 1, group 2 or influenza B HA stalks) but in combination with different head domains from exotic avian influenza virus subtypes^6,7,11^. This leads to preferential induction of responses against the stalk through reactivation of memory B cells, while the de novo response against the novel head domains, which the immune system has never encountered before is relatively weak. We used this approach to perform an observer-blind, randomized, placebo-controlled phase I trial (NCT03300050) to assess the safety and immunogenicity of chimeric hemagglutinin-based vaccines in adults. Results are published^5,6^ and showed that our cHA-based universal influenza virus vaccine approach is safe and elicited a strong response of broadly cross-reactive antibodies against the HA stalk. In addition, the antibody response was durable, and anti-stalk antibodies lasted during the 1.5-year follow-up. These data are encouraging since antibodies directed against the stalk of the group 1 HA have demonstrated not only to provide protection against pandemic H1N1 virus infection^12^, but also disease severity and development of lower respiratory infections^13^. In addition, stalk antibodies have shown antibody-dependent cell cytotoxicity (ADCC) and phagocytosis (ADCP) activity, which can help to clear the virus after the infection has been established in epithelial cells^14,15^. Indeed, the induced antibody response by our cHA group 1 vaccines also showed strong activity in both ADCC and ADCP reporter bioassays^6^.

Here we expand on the safety and immunogenicity studies of our group 1 cHA-based universal influenza virus vaccine candidate^6^ by performing RNA sequencing (RNA-seq) of whole blood from vaccinees to identify gene expression signatures that correlate with the induction of group 1 stalk antibodies. We generated longitudinal unblinded RNA-seq data of 53 volunteers, at early (days 3 and 7) and late (28 days) time points after priming and boosting with the cHA vaccines and found that an early upregulation of genes involved in innate immune response and type I interferon (IFN) signaling was followed by upregulation of genes involved in B cell activation and proliferation in subjects with higher anti-stalk antibody induction. Moreover, cell type deconvolution analysis revealed a significant enrichment for monocytes and different subsets of dendritic cells (DC) that mediated an optimal B cell response. The current study provides a better understanding of the mechanism of regulation of gene expression and the transcriptional pathways that must be activated to induce optimal vaccine responses after vaccination with cHA proteins, which are likely to also be involved in the induction of immunity by other vaccines.

## Results

### Significant changes in gene expression precede the robust induction of anti-stalk antibodies

**Fig. 1:**
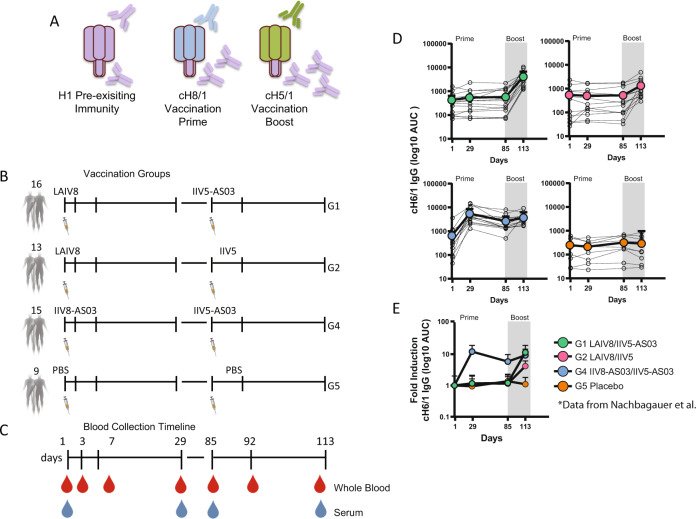
Overview of design and experimental approach (CVIA057; ClinicalTrials.gov identifier NCT03300050). **A** Sequential cHA vaccination strategy and the different vaccination groups. **B** Study design including vaccination and blood collection timeline. Three different vaccination regimens as well as placebo group were included. Group 1 received cH8/1N1 LAIV on day 1 followed by AS03-adjuvanted cH5/1N1 IIV on day 85 (LAIV8-IIV5/AS03). Group 2 received the same vaccination regimen but with the booster vaccination nonadjuvanted (LAIV8-IIV5). Group 4 received adjuvanted cH8/1N1 IIV followed by adjuvanted cH5/1N1 IIV (IIV8/ AS03-IIV5/AS03). Group 5 served as placebo control group and received PBS intramuscularly twice (PBS-PBS). **C** Blood for transcriptomic profiling was collected (indicated in red) at baseline (day 1), days 3, 7, and 29 post prime; and on day 85 (pre-boost), day 93 (day 7 post-boost), and day 113 (day 29 post- boost). Paired samples for antibody quantification (light blue) were collected only on days 1, 29, 85, and 113. **D** Serum anti-H1 stalk titers. IgG titers in the LAIV8-IIV5/AS03, LAIV8-IIV5, IIV8/AS03-IIV5/AS03, and the placebo groups against recombinant cH6/1 HA substrate are shown in Berstein et al.^5^ and Nachbaguauer et al.^6^. The geometric mean titer (GMT, big dots) and confidence interval (CI 95%) are shown. **E** Fold induction during the longitudinal follow up is also shown. Geometric mean fold rise (GMFR, big dots) and confidence interval (CI 95%) are shown.

This clinical trial was designed under the assumption that adult humans possess pre-existing immunity to the H1 HA, including low levels of antibodies and memory B cells with specificity against the HA stalk domain. The objective was to redirect the immune response to the immunosubdominant stalk through sequential vaccination with cHA constructs that feature head domains from avian influenza virus subtypes but share the same H1 stalk domain (Fig. 1A). Fifty-three volunteers were randomized into three different vaccine groups and two placebo control groups in a regimen of prime-booster vaccination: Group 1 (G1) received cH8/1N1 live attenuated influenza vaccine (LAIV) on day 1 followed by AS03A-adjuvanted cH5/1N1 inactivated influenza vaccine (IIV) on day 85 (LAIV8-IIV5/AS03). Group 2 (G2) received the same vaccination regimen but with nonadjuvanted booster vaccination (LAIV8-IIV5). Group 4 (G4) received adjuvanted cH8/1N1 IIV followed by adjuvanted cH5/1N1 IIV (IIV8/AS03-IIV5/AS03). Group 5 (G5) served as an outpatient placebo group and received PBS intramuscularly twice (PBS-PBS). The sequential vaccination strategy, as well as the scheme of vaccination groups and blood collection timeline is shown in Fig. 1A–C. A detailed description of the trial design, immunogenicity and safety of the experimental vaccines can be found in Nachbagauer et al.^6^. Of note, an additional saline intranasally placebo group was enrolled (G3) but was not included for the present RNAseq analysis because it was considered equivalent to G5.

Serum samples for antibody quantification were collected at baseline (day 1), and day 29 post-prime; day 85 (pre-boost) and day 113 (day 29 post-boost) (Fig. 1C). We used data from Nachbagauer et al. ^6^ to represent the anti-stalk antibody induction shown in Fig. 1D, E. The group receiving the IIV8/AS03 priming (G4) had the higher increase in stalk antibodies while priming with LAIV8 did not induce specific antibodies against the stalk of the HA. Comparable results between the groups were found after the boost, with higher induction in subjects from G1 receiving IIV5/AS03 as a booster, followed by volunteers from Group 2 (IIV5 booster). To investigate early molecular changes after receiving the experimental vaccines, whole blood was subjected to RNA sequencing for transcriptional profiling and the correlation of these changes with the anti-stalk antibody responses found in Nachbagauer et al. ^6^ investigated. For this, blood was collected at baseline (day 1), day 3, day 7, and day 29 post prime; and day 85 (pre-boost), day 92 (day 7 post-boost), and day 113 (day 29 post-boost).

**Fig. 2:**
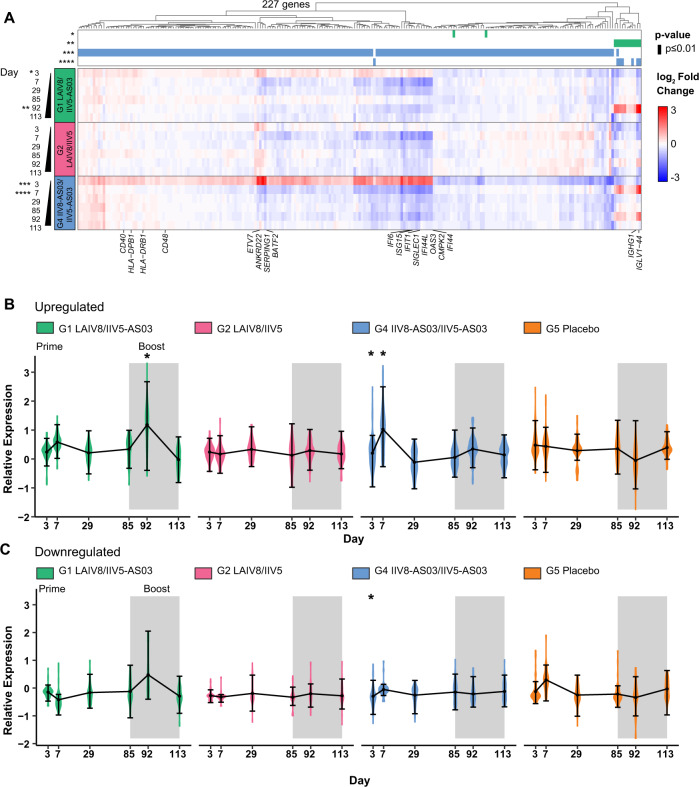
Global transcriptional profile in the CVIA 057 clinical trial. **A** Heatmap of differential expressed genes (DEG) in the groups receiving the experimental vaccine compared to placebo (G5: PBS/PBS): group 1 (G1: LAIV8-IIV5/AS03), group 2 (G2: LAIV8-IIV5) and group 4 (G4: IIV8/ AS03-IIV5/AS03). Average log_2_ fold changes (FC) DEG in each group against placebo are shown at the indicated time points (Bayes adjusted *p* value ≤ 0.05, corrected for multiple testing using the Benjamini-Hochberg. **B**, **C** Temporal expression changes of induced (log_2_FC > 0.25 in at least 50% of the samples) and downregulated genes (log_2_FC < −0.25) related to inflammation pathways GO:0006954 (inflammatory responses), GO:0009615 (response to virus), GO:0034097 (response to cytokines), and GO:0045087 (innate immune response) relative to baseline (day 1) for each of the vaccination groups. Asterisk indicates time points with Bayes adjusted *p* value ≤ 0.05, corrected for multiple testing using the Benjamini-Hochberg (BH) method. The error bars and whiskers indicate mean ± standard error (SE).

We first performed differential gene expression analysis to assess the dynamic changes in gene expression between each of the vaccination groups compared to placebo. A total of 227 genes were differentially expressed between the vaccination groups and placebo for all the timepoints, with no significant differences at baseline. A heatmap of average gene expression changes for each comparison indicated that most changes occurred at day 3 and day 7 in the G4 group (IIV8/AS03 – IIV5/AS03), while significant differences were absent for the other vaccination groups after the prime (Fig. 2A). Although the global transcriptional profile for G1 resembled that of G4, only two genes reached the threshold of significance, *CERK* (*Ceramide Kinase*), and *PRDM8* (*PR Domain Zinc Finger Protein 8*). These two genes were downregulated and are associated with cell migration metabolism during inflammation and negative regulation transcription activity respectively. The initially induced responses decreased by day 28 and pre-boost, at day 85. In addition, we found significantly differentially expressed genes (DEGs) for G1 seven days after receiving the booster dose on day 92. The top G4-induced genes at day 3 included genes related to innate immunity activation and IFN signaling while DGEs at day 7 were related to B cell proliferation signatures. Interestingly, the transcriptional profile was similar between these two groups after receiving the IIV/AS03 vaccine for the first time, marked by expression of *IGHG1*, *IGLV1-44,* and B cell-related genes at day 7 post-prime for G4, and day 7 post-boost in G1. Interestingly, induction of *IGHG1, IGLV1-44* was only present after the use of AS03 adjuvanted formulation, while no induction was found for the non-adjuvanted G2 at day 92 when compared to PBS. Overall, our data could indicate a link between the use of the adjuvant and a specific induction of these genes.

Next, we performed within-group longitudinal comparisons relative to baseline (day 1) levels. This approach allows not only to characterize the transcriptional programs that are being regulated for each vaccination group, but it also controls for interindividual differences in pre-existing transcript levels. When compared to baseline levels within each group, G4 vaccinees showed significant induction or downregulation of at day 3 (2321 genes) and 7 after priming (81 genes), while no changes were detected after the prime dose with LAIV or placebo (G1, G2, and G5, respectively). The longitudinal comparisons also confirmed that after the booster dose, only the subjects who received the IIV5-AS03 (G1) showed significant induction of gene expression (80 genes). Altogether significant transcriptional responses as early as day 3 after vaccination anticipated significant changes in stalk antibody levels measured 29 days later (Table S1).

Transcriptional changes upon immunization with the trivalent LAIV vaccine has previously been reported [PMID: 21743478], given the lack of significant induction in the LAIV8 primed groups we examined the expression changes of inflammation-related genes (gene ontology categories GO:0006954 inflammatory responses, GO:0009615 response to virus, GO:0034097 response to cytokines, and GO:0045087 innate immune response) relative to baseline and regardless of significance for each group (Fig. 2B, C). In agreement with the across-groups comparisons, the longitudinal analysis showed that one of the LAIV8 recipient groups (G1) had a similar trend in temporal gene expression changes as G4 during the priming. We then performed Gen Set Enrichment Analysis (GSEA) for the same longitudinal comparisons for which both, innate and adaptive immune pathways were enriched after the priming LAIV8 dose in G1 and to a lesser extent in G2 (Figure S1).

### IIV8/AS03 – IIV5/AS03 administration induced early activation of cell signaling and innate immune pathways associated with B cell proliferation and induction of anti-stalk antibodies

**Fig. 3:**
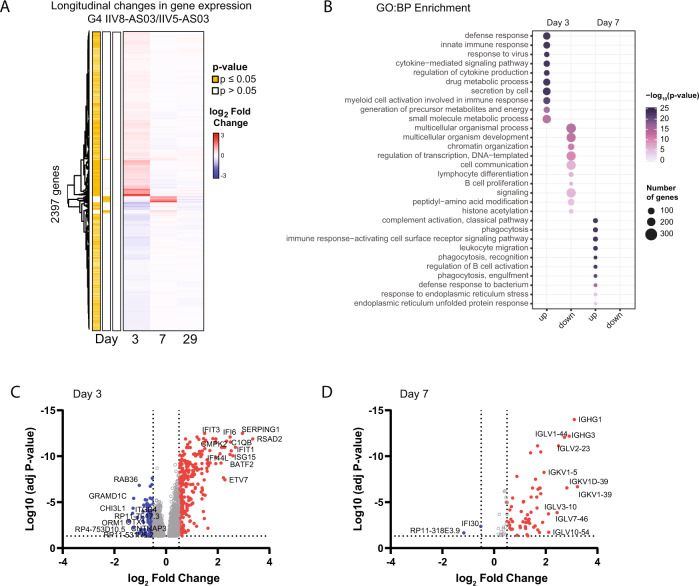
Longitudinal transcriptional profile of IIV8/AS03-IIV5/AS03 vaccination group (G4). **A** Heatmap of DEG changes on day 3, 7, and 29 of subjects of G4. Average fold change relative to baseline levels is shown and statistically significant differences are highlighted in yellow (Bayes adjusted *p* value ≤ 0.05, corrected for multiple testing using the Benjamini-Hochberg (BH) method). **B** Gene ontology (GO) enrichment analysis of differentially expressed genes (DEGs) on day 3 and 7 priming with IIV8/AS03. Diagram shows the top 10 enriched biological processes. Gene ontologies are ranked by their significance and dot size indicates overlap on number of genes. **C**, **D** Volcano plots of upregulated (red) and downregulated (blue) genes. Expression differences > 1.5-fold change (FC) are highlighted (log_2_FC ≤ 0.5, vertical dotted lines). The names of the top 10 genes are shown. Source data referring to A-B are provided as a Source Data file.

To better understand the relationship between anti-stalk antibody induction and the dynamic regulation of gene expression longitudinally, we expanded our previous analysis and investigated the functional pathways perturbed after prime-boost in G4 (IIV8/AS03 – IIV5/AS03) vaccinees. We first analyzed changes early after priming (day 3 and day 7). As shown in Fig. 3A, significant differences were mostly found on day 3, with 1317 upregulated genes versus 1004 genes downregulated. The number of upregulated genes decreased on day 7 (80 genes) and only two genes were found to be downregulated. By day 29, no significant differences were found relative to the vaccine regimens suggesting that gene expression changes were back to baseline. We next used this analysis to perform gene ontology (GO) enrichment analysis (Fig. 3B) and to build volcano plots (Fig. 3C, D). The gene expression changes were associated with 59 (up-) and 33 (down-) biological processes (BP) categories on day 3 (Source Data 1), which included innate immune responses and responses to virus infection. Particularly, the top-10 enriched GO categories of induced genes included cytokine signaling and immune cell activation (Fig. 3B). Genes included in these pathways were classical genes induced in response to virus exposure, such as *ISG15*, *IFIT3*, *OAS1*, *DDX58* (RIG-I) or *SERPING1*, among others (Fig. 3C and Table S1). In contrast, downregulated BP included chromatin organization and regulation of transcription and histone acetylation, suggesting cell remodeling changes triggered by immune signaling. On day 7, the top-10 GO categories of upregulated genes transitioned from innate immunity to activation of adaptive immune cells (Fig. 3B), including leukocyte migration, phagocytosis and recognition, and regulation of B cell activation. In total there was enrichment of 17 GO categories on day 7 post-prime (Table S1), aligned with a high upregulation of immunoglobulin transcripts and the *CD38* cell activation marker (Fig. 3D and Table S1). When looking at the top upregulated immunoglobulin transcripts, we found that the response was dominated by a variety of clonotypes, with an overrepresentation of *IGKV1-39* followed by *IGHG1* > *IGLV1-44* > *IGKV1D-39* > *IGHG3* (Fig. 3D). As expected, by day 29 gene expression reverted to pre-vaccination levels and no significant differences were found when the peak of the antibody response was detected by serological methods. Similarly, no significant changes of the transcriptional profile were detected on day 85, before the booster dose, or on day 93 (post-boost), consistent with a minimal increase of anti-stalk antibodies in this group (Fig. 1C).

**Fig. 4:**
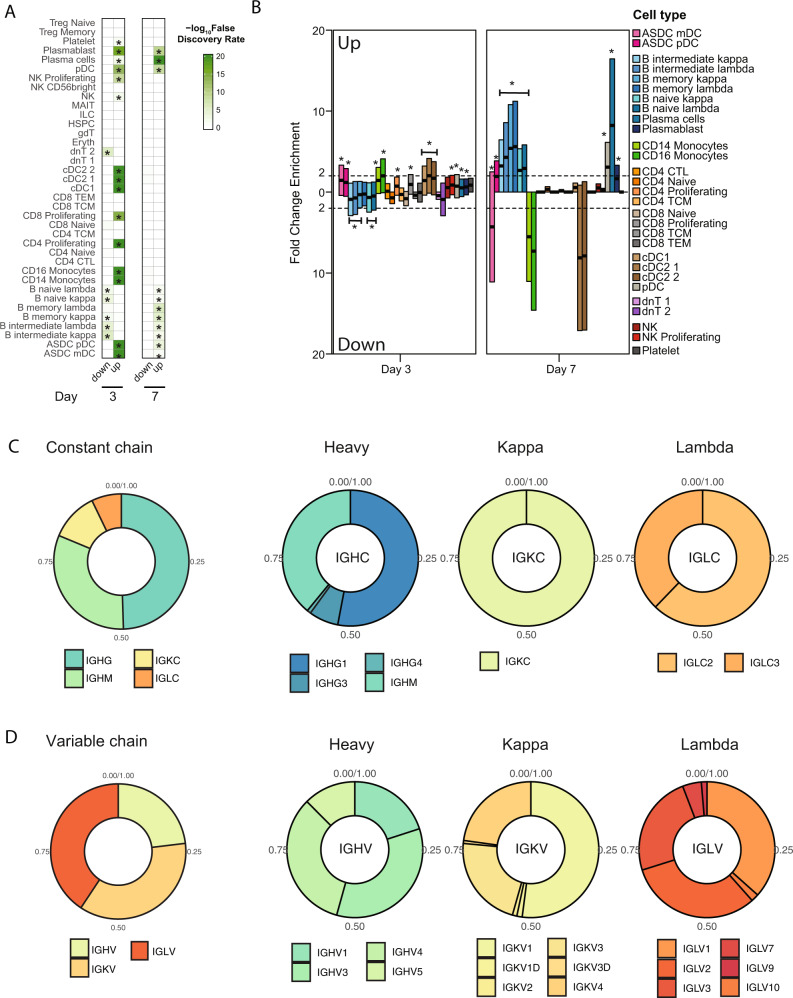
Cell type deconvolution and induction of immunoglobulin genes in G4. **A** Main cell types enriched or depleted in the peripheral blood of patients after priming with IIV8/AS03 compared to baseline (day 1) on day 3 and 7. Asterisk indicates FDR < 0.05 (Fisher’s exact test, cell type reference from ref. ^16^). **B**) Fold change enrichment of intersected gene sets of G4 after cell type deconvolution on day 3 and 7. Bar indicates fold change enrichment (up) or depletion (down) for each cell type on day 3 and 7. Asterisk indicates FDR < 0.05 (Fisher’s exact test). **C**, **D** Relative abundance of immunoglobulin transcripts on day 7 after priming with IIV8/AS03. Relative proportion of enriched isotypes and subclass transcripts corresponding to VJ recombination is shown for the constant (**C**) and the variable (**D**) heavy, kappa, and lambda chains (IGH, IGK, and IGL).

The immune response is a complex biological process that requires multiple interactions among immune cells with heterogenous differentiation states and signaling pathways. We next decided to interrogate the changes in function and cell-type composition of the cells circulating in blood after vaccination with the cHA influenza vaccine. For this we used transcriptional signatures obtained from healthy peripheral blood mononuclear cells (PBMCs) profiled by bimodal protein-RNA measurements with Cellular Indexing of Transcriptomes and Epitopes by Sequencing (CITE-Seq) at the single-cell level^16^. These cell type-specific gene expression signatures consisted of 58 cell types and subtypes, including subsets of the T, B, and myeloid cell compartments. To simplify cell type groups, subclusters were collapsed into 37 major cell types. We applied a false discovery rate (FDR) < 0.05 (adjusted *p* value, Fisher’s exact test) to deconvolute the bulk DEG data and determine cell type-specific specific signatures enriched or depleted in the peripheral blood of G4 after priming with IIV8/AS03 on day 3 and 7 (Fig. 4A). Fold-change gene signatures revealed a significant enrichment for monocyte markers, both classical-CD14+ and non-classical-CD16+, and different markers of dendritic cell (DC) subset on day 3 post-prime. To note, from our bulk RNAseq data, we are not able to distinguish between enrichments due to changes in the relative composition of blood cell types versus enrichments due to functional activation of immunological cell states and signaling pathways in such cell types. Regardless of this limitation, the results were consistent with the biological processes found to be enriched in the GO analysis. The enriched signatures are associated with populations that are main mediators of cell signaling processes, innate immune responses, and antigen presentation. Enrichments on day 7 suggested that relative cell type-specific signatures and/or composition changed to an enrichment of B cells with different maturity state: memory > intermediate > naive of the kappa and lambda chain expressing cells. At this timepoint a significant enrichment was also found for antibody-secreting cells: plasma cells > plasmablast suggesting the presence of affinity matured long- and short-life antibody-producing cells induced by the universal vaccine prototype. In addition, plasmacytoid DCs (pDCs) specific signatures were also induced at days 3 (*p* value = 1.04 × 10^−13^, Fisher’s exact test) and 7 (*p* value = 4.09 × 10^−9^), with the earlier enrichment of conventional DC (cDCs), on day 3 post-prime. Figure 4B shows the fold-change enrichment for 29 cell types, including subsets from the main cell type lineages: CD4+ and CD8+ T cells, B cells, DCs, monocytes, and natural killers (NK) on day 3 and 7 post-prime. An additional analysis with a different enrichment method (xCell [PMID: 29141660]) and cell-type reference showed that most of the results from our original cell type enrichment analysis were consistent, with a few differences. In particular, pDCs were only enriched at day 3 and not at day 7, and plasma cells were not enriched at day 3 but only at day 7 (Fig. S2). Finally, we quantified the diversity of immunoglobulin (Ig) transcripts that were differentially expressed, as a proxy for the induction of antibodies in response to vaccination. The relative composition of enriched isotypes and subclasses, and the heavy, kappa light, and lambda light chain (IGH, IGK and IGL) loci usage for the variable (V) regions on day 7 after priming with IIV8/AS03 are shown in Fig. 4C and D. Enriched transcripts included members of all loci (Fig. 4C). Much of the response of the constant (C) region was dominated by IGHG and IGHM isotypes, while the most abundant subtypes were, as expected, *IGHG1* > *IGHG3* > *IGHG4* (Fig. 4C). For the V regions, the majority of IGH genes were from the *IGHV3* (35%) and *IGHV4* (32%) subgroups followed by *IGHV1* > *IGHV5*. For the IGK locus, enriched transcripts were dominated by those from the *IGKV1* (51%) and *IGKV3* (24%) and *IGKV4* (23%) subgroups, while the IGL locus was dominated by *IGLV1* (36%) followed by *IGLV2* (33%) and V3 (24%), and included transcripts from at least 6 different loci^17^ (Fig. 4D).

### Early and strong induction of the antiviral response correlates with high anti-stalk antibody titers

**Fig. 5:**
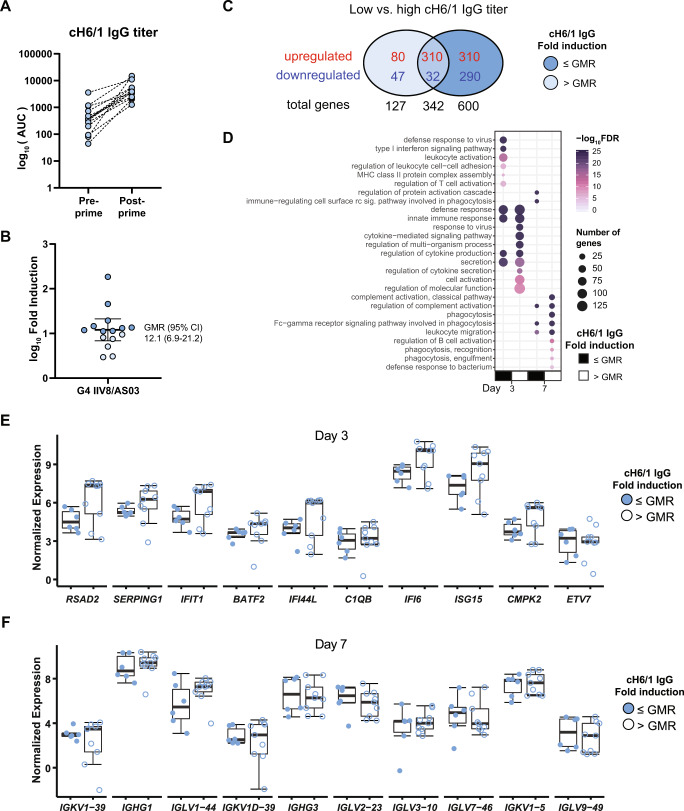
Transcriptional changes in subjects with high induction of anti-stalk antibodies in G4 after priming with IIV8/AS03. **A** IgG titers against cH6/1 in group one subjects after prime. Figure shows titers at baseline (day 1) and day 29 after vaccination with IIV8/AS03. **B** Fold induction after vaccination is also shown. Geometric mean rise (GMR) titers were calculated and GMR (95% CI) is indicated. Black bar indicates GMR values, box indicates IQR (Q1–Q3), lines indicate minimum and maximum. **C** Venn diagram showing the number of DEGs between subjects with low (≤GMR, light blue) or high (>GMR, dark blue) fold induction of anti-stalk antibodies at day 3 and 7 after prime. **D** Gene ontology (GO) enrichment analysis of differentially expressed genes (DEGs) at day 3 and 7 after priming with IIV8/AS03 in subjects with ≤GMR fold induction compared to subjects with fold induction >GMR. The top 10 enriched GO:biological processes are shown ranked by their significance, and dot size indicates overlap on number of genes. The GO enrichment was performed with a ranked query and multiple testing analytical correction, FDR ≤ 0.05 or −log_10_FDR ≥ 0.3. **E**, **F** Normalized expression of top 10 genes at the indicated time points in subjects according to anti-stalk antibody induction relative to the GMR titers. Black bar indicates median values, box indicates IQR (Q1–Q3), lines indicate minimum and maximum. Source data referring **E**, **F** are provided as a Source Data file.

Next, we asked whether individual differences of the induction of specific antibodies targeting the stalk of the HA of group 1 influenza viruses after vaccination with IIV8/AS03 – IIV5/AS03 (G4) could be linked to specific and unique transcriptomic signatures. First, we looked at individual cH6/1 IgG antibody responses before (pre-) and 29 days (post-) after prime. All vaccinees showed similar antibody titers post-prime (Fig. 5A). We next calculated the fold rise on cH6/1 antibodies as the ratio between post- antibody value to pre-levels for each vaccinee. We then computed the geometric mean rise (GMR) by taking the exponent (log_10_) of the mean fold rise of all individuals. As shown in Fig. 5B, GMR (95% CI) was 12.1 (6.9–21.2). We then set a GMR value of 10 as a threshold of higher (>GMR, above the geometric mean) versus lower (≤GMR, below the geometric mean) vaccine responders. A comparison of both low (*n* = 6) and high (*n* = 9) responders at baseline showed no pre-existing differences that could explain the variability in the magnitude of the antibody response after vaccination. We next investigated differences on gene expression on day 3 and 7 post-vaccination by comparing each group to their respective baselines (day 0). A total of 469 (390 up and 79 down) and 942 (620 up and 322 down) genes were differentially expressed for the low and high responders, respectively, on day 3. Among these genes, 310 were upregulated in both groups, all of them related to canonical immune response processes (Fig. 5C, D). Gene ontology analysis of genes that were exclusively up (*n* = 80) in the low responders returned no significant GO annotated processes, whereas those that were exclusively up in the high responders included additional genes mainly related to innate immune response and cytokine-mediated signaling (Fig. 5D, top 10 categories). In addition, processes exclusively upregulated in high responders included mitochondrial organization, respiratory chain and upregulation of transcription factor activity (Source Data 2). For the downregulated genes, those that were common or exclusive to either group did not return relevant GO categories implicated in immune activation (Source Data 2). We then compared levels of the top-10 expressed genes between individuals with low and high antibody titers. Results showed that, while non-significant, expression across the top DEGs was lower in individuals with fold induction of anti-stalk antibodies below the geometric mean (Fig. 5E). While differences in group size (low, *n* = 6 vs high *n* = 9) could affect the power to detect DEGs, the differences in absolute expression levels in each group suggest that the overall differences between low and high responders were due to the magnitude of the induction rather than the expression of specific gene programs. On day 7, the transcriptional response for both low- and higher-responders converged in the expression of an adaptive immune response signature, including complement activation, leukocyte migration and B cell activation (Fig. 5D). Notably, the levels of expression for Ig genes on day 7 were similar regardless of the magnitude of the measured antibody response (Fig. 5F).

### G1 and G4 induce similar functional patterns but different immunoglobulin repertoires

**Fig. 6:**
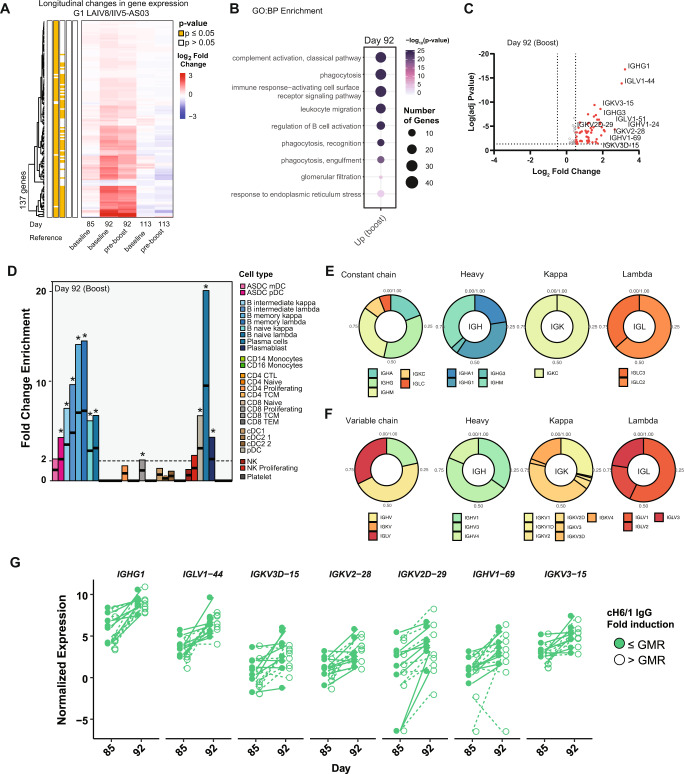
Transcriptomic profile of LAIV8-IIV5/AS03 vaccination group (G1) on day 92 (7 days after the booster dose). **A** Heatmap of longitudinal DEG changes at day 92 (day 7 after boost) and day 113 (day 29 after the boost) of subjects in G1 compared to day 1 before priming with LAIV87 and after priming on day 85. Average log_2_ fold change from baseline and on day 85 are shown and statistical differences are highlighted in yellow on the left (Bayes adjusted *p* value ≤ 0.05, corrected for multiple testing using the Benjamini-Hochberg (BH) method). **B** Gene ontology (GO) enrichment analysis of differentially expressed genes (DEGs) on day 7 after boost. Diagram shows top 10 enriched biological processes ranked by their significance, and dot size indicates overlap on number of genes. The GO enrichment was performed with a ranked query and multiple testing analytical correction, FDR ≤ 0.05 or −log_10_FDR ≥ 0.3. **C** Volcano plots of upregulated (red) and downregulated (blue) genes. Differences >1.5-fold are highlighted (vertical dotted lines). The names of the top 10 genes are shown. **D** Fold change enrichment for cell-type specific gene sets in G1 at day 7 after boost with IIV5/AS03. Bar indicates fold change enrichment (up) or depletion (down) for each cell type after deconvolution. Asterisk indicates FDR < 0.05 (Fisher’s exact test). **E**, **F** shows relative proportion of enriched isotypes and subclass transcripts corresponding to VJ recombination is shown for the constant (**E**) and the variable (**F**) heavy, kappa and lambda chains (IGH, IGK, and IGL). **G** Dotplots of expression of the top 10 upregulated genes in G1 from day 85 to 92 according to fold induction of anti-stalk antibodies in serum (empty dot > GMR, filled dot ≤ GMR). GMR Geometric mean rise.

As shown before, priming with LAIV8 did not induce specific antibodies against the stalk of the HA protein (Fig. 1C). However, LAIV-primed individuals showed a significant increase of antibody titers after boosting with IIV5/AS03, or IIV5 only, in the G1 and G2 groups, respectively. We aimed to characterize the functional pathways perturbed by the booster regimen. We interrogated G1 first since antibody responses on day 113 (29 post-boost) were higher compared to the other vaccination groups after the booster dose. Longitudinal changes of gene expression were determined after normalization by baseline or day 85 (pre-boost) as indicated in Fig. 6A: day 85, 92, and 113 using baseline as a reference; or day 92 and 113 using day 85 as a reference. No significant differences were found between baseline before priming (day 1) and pre-boost (day 85), and DEGs at day 92 showed comparable transcriptomic profiles independent of the reference used. However, a higher number of significant DEG’s compared to baseline than to day 85 was found. Enrichment of 72 genes related to B cell activation and immunoglobulin transcription were identified: *IGHG1* > *IGLV1-44* > *IGKV2D-29* > *IGHV1-69* > *IGKV2-28*. Remarkably, similarly than G4 after prime, induction of *IGHG1, IGLV1-44* was high, and only present after the use of AS03 adjuvanted formulation. Furthermore, we found preferential usage of *IGHV1-69* among the top-10 DEGs. In addition, *IGHV1-69-2*, and *IGVH1-18* were also found among the top-25 genes induced after the boost. Since anti-HA stalk antibodies are preferentially encoded by immunoglobulin heavy chain V region gene VH1-69 and VH1-18^18^, usage of these clonotypes indicated that the vaccine used in this trial induced specific and not generic immune responses against the stalk of group 1 HA protein. The intersection of the top-10 most significant biological processes, and top-10 significant genes at day 92 are shown in Fig. 6B, C. Finally, gene expression levels at day 113 (day 29 post-boost) were restored to baseline levels before vaccination, similar to those on day 29 after prime.

Before we showed that G1 (LAIV8-IIV5/AS03) and G4 (IIV8-IIV5/AS03) induced similar antibody levels and functional patterns after receiving the inactivated adjuvanted cHA vaccine for the first time, either after boosting or priming, respectively (Fig. 2A). Since G1 vaccinees were primed with LAIV8, we asked whether relative cell type composition and relative Ig repertoire would also be the same. Cell type deconvolution (shown in Fig. 6D) indicated similar enrichment of cell types enriched in G1 after boost compared to G4 after prime (Fig. 4A). However, some differences were found when comparing the Ig transcripts encoding for the C region of immunoglobulin heavy chains. Ig alpha (IgA) is the major immunoglobulin class in body secretions and can be found on linings of the respiratory tract, digestive system, and saliva^19^. It was expected that the administration of LAIV8 would induce mucosal antibodies at the mucosal surfaces. However, saliva and serum samples analyzed by enzyme-linked immunosorbent assays (ELISAs) for anti-stalk IgA and secretory IgA (sIgA) showed no such antibodies in G1 or G2 after LAIV administration^6^. Contrary to this, when we look at the relative abundance of the Ig repertoire transcripts (Fig. 6E, F), we found that it was still dominated by IGHG, but vaccinees from the G1 also showed an enrichment of the IGHA subclass (19%). This contrasted with G4, in which the response was dominated by IGHG followed by IGHM and no IGHA transcripts were detected (Fig. 4C). Except for the presence of IGHA1 transcripts, the composition of transcripts from the C region in G1 (Fig. 6F) was similar to G4 (Fig. 4D). However, the diversity and the relative proportion of Ig transcripts at the V chains from the IGH, IGK, and IGL loci was also different from those elicited by the G4. Subgroups of encoded transcripts for G1 included *IGHV3* (46% each), *IGHV1* (35%), and *IGHV4* (19%) only, whereas the IGK responses were dominated by the *IGKV3* subgroup (*IGKV3* and *IGKV3D* accounted for 45% of *IGKV* detected transcripts) followed by the *IGKV3* (29%) and *IGKV2* (6%). Lastly, the induced IGL transcripts were less diverse compared to G4, with only IGLV1 (57%), *IGLV3* (23%), and *IGLV2* (20%) subgroups being expressed. The immunoglobulin repertoire on day 7 after boost with IIV5/AS03 across the differentiation spectrum is shown in Fig. 6E. The observed differences in the repertoire of Ig transcripts and detection of IgA expression show that the intramuscular adjuvanted vaccine effectively boosted previously undetectable mucosal immunity induced by the LAIV8 prime. This in contrast with G2 for which the only difference in the vaccine regime was the presence of adjuvant in the booster dose. To understand the possible contribution of the adjuvant on the induction of IGHA1 and IGHA2, we also investigated levels of *IGHA1* or *IGHA2* in G4 subjects who received IIV8/AS03-IIV5/AS03. After comparison with baseline levels (Day 1) or 7 days post-boost (Day 92), no enrichment of *IGHA1* or *IGHA2* was found when only inactivated-adjuvanted formulation was used. This indicates that while adjuvant can be related to the increase of IGHA1 or IGHA2 when priming with LAIV8, no effect on IGHA1 or IGHA2 is observed when only inactivated-adjuvanted formulation was used.

Finally, similar to G4, we looked at expression levels across individuals that developed low (≤GMR) or high (>GMR) cH6/1 antibody titters, as measured by ELISA. Both groups showed similar levels of gene expression on day 7 post-boost, regardless of measured antibody titers at the late timepoint post-boost, on day 113 (Fig. 6G). This was independent of pre-existing immunity as Ig transcript levels before priming or boosting showed no correlation with induction of antibody levels.

### Differences of vaccine-induced antibody responses attributed to the adjuvant

**Fig. 7:**
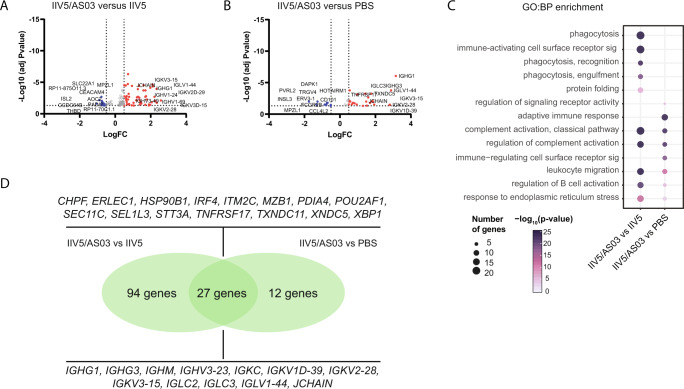
Contribution of AS03 adjuvant to gene expression changes. **A** Volcano plots of upregulated (red) and downregulated (blue) genes in adjuvanted G1 (IIV5/AS03 booster) vs non-adjuvanted G2 (IIV5) post boost. Differences higher than 1.5-fold are highlighted and names for the top 10 genes are shown. **B** Volcano plots of upregulated (red) and downregulated (blue) genes in adjuvanted G1 vs placebo (G5) groups. **C** Gene ontology (GO) enrichment analysis of differentially expressed genes (DEGs) at day 7 after boost for the indicated comparisons. Diagram shows top 10 enriched biological processes. Gene ontologies are ranked by their significance and dot size indicates overlap on number of genes. The GO enrichment was performed with a ranked query and multiple testing analytical correction, FDR ≤ 0.05 or −log_10_FDR ≥ 0.3. **D** Venn diagram of DEGs for the comparison between G1 (IIV5/AS03 booster) versus G2 (IIV5), and G1 (IIV5/AS03 booster) versus G5 (placebo) at day 92.

Because we observed that higher IgG responses in serum were associated with the use of the AS03 adjuvant after boosting with the inactivated formulation, we interrogated the specific contribution of the adjuvant to the gene expression signatures detected. For this, we compared DEGs between G1 (IIV5/AS03) versus G2 (IIV5) on day 7 post-boost in parallel to DEGs between G1 (IIV5/AS03) and G5 (PBS) at the same time point. Analysis showed 92 (up-) and 28 (down-) genes for the G1 vs. G2 comparison; and 29 (up-) and 10 (down-) for the G1 vs. placebo (Fig. 7A, B). When we compared the GO categories enriched, we determined that both comparisons shared categories such as complement activation (classical pathway), leukocyte migration, and regulation of B cell activation. In contrast, some categories were unique for the G1 vs. G2 including phagocytosis (recognition and engulfment), immune-activating cell surface receptor signaling pathway or protein folding (Fig. 7C). Finally, we considered that the comparison of G1 versus G2 assessed the contribution of the adjuvant independently (although in the context) of IIV5, while the comparison of G1 versus G5—placebo—assessed the contribution of both IIV5 and AS03. If we then intersect the datasets obtained, the induction or depletion of those genes in common should be driven by the use of the AS03 adjuvant independently. As shown in the Venn-diagram (in Fig. 7D), 27 of the induced genes were shared between both datasets. While 12 of them were directly related to transcription of Ig: *IGHG1*, *IGHG3*, *IGKV1D-39*, or *JCHAIN* among others; 15 referred to other processes such as regulation of innate and adaptive immune responses, including complement activation, response to stress, and B cell development: *IRF4*, *XBP1*, *ITM2C*, *MZB1*, *PDIA4*, *POU2AF1*, *STT3A*, *TNFRSF17* (Supplemental Table 1).

## Discussion

Universal influenza virus vaccines are urgently needed. We have carried out a phase 1 clinical trial to test the immunogenicity of a chimeric HA-based (cHA) group 1 universal influenza virus vaccine and showed that the cHA vaccine was safe and induced high anti-stalk antibody titers with broad activity against group 1 HAs (as reported in Nachbagauer et al.^6^. In particular, subjects vaccinated with adjuvanted, inactivated cHA vaccines at either prime or boost -IIV8/AS03 or IIV5/AS03, respectively- induced high anti-stalk antibody titers^6^. To further our understanding, we used a systems biology approach and performed longitudinal transcriptomics analyses after both prime and boost with the universal influenza virus vaccine prototype.

While the transcriptional changes that occur during influenza vaccination and infection have been previously described^20,21^ some of the early studies were performed with microarrays (using predefined sets of immune-related transcripts), in the context of conventional subtype/strain-specific vaccines, or enriched for specific cell types^22–24^. For this study we generated global transcription profiles from whole-blood bulk RNAseq data and show that gene expression changes associated with two universal vaccine regimes G1 and G4 (IIV8/AS03-IIV5/AS03 and LAIV8-IIV5/AS03) results in induction of cell composition changes and gene programs that correlate with robust antibody responses, in this case to anti-stalk epitopes. In a previous study with conventional TIV and LAIV influenza vaccines, Nakaya et al. [PMID: 21743478] reported that TIV-induced transcriptional signatures were better correlated with antibody titers compared to LAIV, where in contrast to our study LAIV priming resulted in significant changes in gene expression. In our study priming with LAIV8 showed a modest induction (below the level of significance) for G1 and G2, with significant enrichment of inflammatory pathways by GSEA analysis. The reasons for the lack of statistical support in our study could be due to smaller group sizes, differences in vaccine dose and composition, or differences in immunogenicity of the antigens used.

This study complements and adds on our previous profiling of serological and mucosal anti-stalk vaccine responses^25^ by dissecting the cellular composition of the immune response induced by the different vaccination schemes. As shown by Nachbagauer et al., immunogenicity for G1 and G4 (IIV8/AS03-IIV5/AS03 and LAIV8-IIV5/AS03) was similar at the protein level, however, differences in diversity observed for the Ig transcripts between these groups suggest that these two groups may have developed different Ig repertoires with yet unknown roles in response to infection.

Our study also provides the transcriptional context of immune responses that lead to the development of broadly neutralizing antibodies specific to the HA stalk in the same individuals^25^. When we compared transcriptional responses according to vaccination groups, our data demonstrated that subjects with optimal stalk antibody responses showed an early upregulation of genes involved in innate immune response and type I IFN signaling. As expected, triggering the innate immune compartment corresponded to a robust upregulation of genes involved in B cell activation and proliferation in subjects from G4. In addition, we found a number of consistently enriched blood cell type-specific signatures associated with these processes. An early enrichment of monocytes, and different subsets of dendritic cell (DC) cells was especially marked on day 3 reflecting innate immune activation, while cell type composition transitioned to B cell responses on day 7. Importantly, our analyses suggest the transient enrichment of long-lived plasma cells in peripheral blood post vaccination, which is a desirable quality for any vaccine^26,27^. However, the accurate identification of cell type-signatures in our RNAseq data is subject to the accuracy of the reference data set used to deconvolute this data, and the presence of the different cell subtypes should be confirmed by alternative methods. While not surprising, these results support the inclusion of immunomodulatory components as a means to improve vaccine performance and even in to reinforce mucosal immunity^28–30^. Adjuvanted-vaccine recipient groups were not only the ones that show significant changes in gene expression in response to vaccine administration, but we also found that differences of transcriptional programs were highly dependent on the use of the adjuvant. In particular, vaccinees boosted with IIV5/AS03 (G1) showed a unique and distinct upregulation of biological processes directly related to activation and developmental programs of the immune system when compared to IIV5 only (G2). For example, XBP1 and IRF4 were correlated with the use of the adjuvanted vaccine. These two transcription factors are essential in the differentiation of activated B cells into plasma cells^31–34^. XBP1, in particular, is part of the endoplasmic reticulum (ER) stress response, the unfolded protein response (UPR), and well known for its role not only on B-cell expansion, maturation, and class switching of antibodies for an optimal immunoglobulin production, but also almost every immune cell type^35–38^. In contrast, IRF4 is essential for plasma cell survival^39^. The apparent differences found in the induction of such factors in the adjuvanted vaccine group compared to IIV5 only, reflects the direct activation of immune compartments at multiple cell levels by the AS03-formulated vaccines, which is likely to be related to the molecular mechanisms associated with AS03 adjuvanticity. To note, no samples were collected at day 3 post-boost, therefore some limitations have to be acknowledged on the use of adjuvant after booster.

In addition, we also investigated the gene signatures associated with the magnitude of the antibody responses. This hasn’t been previously shown for universal influenza vaccines, and it is important since a stalk antibody titer of 10^3^ and 10^4^ can suffice to prevent and reduce the severity of influenza virus infection, respectively^12,13^. Importantly, the anti-stalk antibody titer after vaccination with the cHA in this trial exceeded those levels. After comparison of high (>GMR) versus low (≤GMR) responders in G4, we found that individuals with greater induction did not have an independent transcriptomic profile, but rather higher levels of early-induced signatures of inter-related biological pathways on day 3, which promoted an optimal immune response. On the other hand, levels of expression for immunoglobulin genes were similar regardless of the magnitude of the measured antibody response. Since this study was not designed to address within vaccine-group differences, differences found in the high versus low responders have to be cautiously interpreted. Nonetheless, this trend suggests that more granular analyses and fine-tuning of early responses (day 1 to 3 post-vaccination and post-booster) might be crucial to define and understand vaccine-correlates of protection. Nonetheless, both high and low responders showed specific and not generic usage of immunoglobulin transcripts such as *IGHV1-69* or *IGKV1-39* frequently used by stalk-binding heterosubtypic antibodies^18,40–42^. Finally, our study also allowed for the simultaneous detection of the overall transcriptional responses of immunoglobulins within the transcriptome of the vaccinated volunteers. Profiling of the Ig repertoire in G4 after vaccination showed primary and secondary responses with induction of both IgG and IgM isotypes. More importantly, different subtypes could be distinguished, with high frequency of *IGHG1* and *IGHG3*, classically related to the higher potency of antibody-mediated functions, such as ADCC or complement activation. In contrast, *IGHG2*, typically related to response to bacterial infections, was not induced. Another key observation was the detection and observed enrichment of IGHA transcripts, particularly *IGHA1*, after boosting with IIV5/AS03 (G1) in subjects primed with LAIV. This is relevant since IgA responses were not detectable in serum or on mucosal surfaces after any of the vaccine doses and demonstrate that system biology approaches, such as transcriptomics, can provide a comprehensive picture of how the immune system responds to vaccination. Nonetheless, it is still possible that some IgA was induced, but levels were under the limit of detection of the ELISA used for quantification in this trial. On the other hand, no nasal washes samples were collected to measure IgA after LAIV8 administration. The subtle effect of priming with the LAIV is also supported by the longitudinal DEG changes at day 92 post-boost in G1 (LAIV8/IIV5-AS03). A higher number of significant DEG’s compared to baseline than to day 85 was found, indicating a concealed effect of the prime dose. Further studies will help to understand the necessary pathways that need to be triggered to induce anti-stalk antibodies after vaccination with cHA influenza vaccines in combination with different vaccine regimes. Such insight can be exploited to develop effective universal influenza virus vaccines.

## Methods

### Study design

A randomized, placebo-controlled, observer-blind, phase I clinical study was conducted at Cincinnati Children’s Hospital Medical Center (Cincinnati, OH, USA) and Duke Early Phase Clinical Research Unit (Durham, NC, USA). This clinical trial was designed to assess the safety and immunogenicity of a chimeric hemagglutinin-based vaccine approach; and its ability to elicit broadly cross-reactive antibodies against the hemagglutinin stalk domain of influenza viruses. The trial design has been previously published^5,6^. Briefly, 66 participants were block-randomized in each site and received LAIV8-IIV5/AS03 (Group 1, G1), LAIV8-IIV5 (Group 2, G2), SALINE-PBS (Group 3, G3), IIV8/AS03-IIV5/AS03 (Group 4, G4) or PBS-PBS (Group 5, G5). To note, LAIV8 was administered intranasal while the IIV5 and IIV5/AS03 vaccines were given intramuscularly. The Cincinnati Children’s Hospital Medical Center Institutional Review Board (IRB) served as the central IRB of record for review, approval and oversight of this study on behalf of the Icahn School of Medicine at Mount Sinai IRB, Duke IRB, and PATH Research Ethics Committee. All patients provided written informed consent prior to participation. ClinicalTrials.gov identifier NCT03300050. De-identified samples were made available for this analysis (Icahn School of Medicine at Mount Sinai IRB approval #IRB-17-01779).

### Study participants

The study was carried out between 10 October 2017 and 9 August 2019. General inclusion criteria were male or non-pregnant female between, 18 and 39 years at the time of the first vaccination. Exclusion criteria included the previous history of Guillain-Barré syndrome, immunosuppression, history of influenza virus vaccination within 6 months prior to study enrollment or use of any other investigational drug or vaccine other than in the present study, among others. A complete list of inclusion and exclusion criteria is provided at https://clinicaltrials.gov/ct2/show/NCT03300050.

### Enzyme-linked immunosorbent assay (ELISA)

The methods and results of antibody responses against the stalk domain of the HA protein of influenza group 1 viruses used in this study have been previously published^6^.

### RNA isolation, library preparation, and sequencing

PAXgene blood samples were processed for total RNA extraction using the Agencourt RNAdvance Blood Kit (Beckman Coulter) on a BioMek FXP Laboratory Automation Workstation (Beckman Coulter) according to the manufacturer’s instructions. Concentration and RNA integrity number (RIN) of isolated RNA were determined using Quant-iT™ RiboGreen™ RNA Assay Kit (Thermo Fisher) and an RNA Standard Sensitivity Kit (DNF-471, Agilent Technologies, Santa Clara, CA, USA) on a Fragment Analyzer Automated CE system (Agilent Technologies), respectively. Subsequently, RNA-seq libraries were constructed from 300 ng of total RNA using the Universal Plus mRNA-Seq kit (Tecan Genomics, San Carlos, CA, United States) in a Biomek i7 Automated Workstation (Beckman Coulter). The transcripts for ribosomal RNA (rRNA) and globin were further depleted using the AnyDeplete kit (Tecan Genomics) prior to the amplification of libraries. Library concentration was assessed fluorometrically using the Qubit dsDNA HS Kit (Thermo Fisher), and quality was assessed with the Genomic DNA 50Kb Analysis Kit (DNF-467, Agilent Technologies). Preliminary sequencing of the libraries was performed using a MiSeq system (Illumina) to confirm library quality. Deep sequencing was subsequently performed using an S2 flow cell in a NovaSeq sequencing system (Illumina) (average read depth ~30 million pairs of 2 × 95 bp reads) at the New York Genome Center.

### RNA-sequencing analysis

Illumina’s Real-Time Analysis (RTA) software was used for base-calling and quality scoring of RNA-sequencing (RNA-seq) data. Sequencing reads were processed and mapped to the human hg38 reference genome (Release 23 GRCh38.p3) with custom analysis scripts that combine publicly available tools as described before^43,44^. A combined matrix of mapped paired end read raw counts (genes x samples) was then obtained with featureCounts^45^ and used as input for differential gene expression (DGE) analysis in R v3.6.2. Prior to DGE analysis, gene counts were normalized to fragments per kb per million reads (FPKM) with RSEM with default settings for strand-specific data^46^. Genes with <1 FPKM in at least 50% of samples were removed from the analysis. Next, normalization factors were estimated using the trimmed mean of M-values (TMM) method, followed by voom mean-variance transformation^47^ to account for differences in coverage across samples. The data were inspected for potential confounders including the variables sex, site where sample was collected, RNA extraction batch and input RNA concentration, with Limma linear modeling^48^. Only sex was fitted as a covariate in the final model using a per-patient block design. Pairwise comparisons of each vaccine group (G1, G2, and G4) were performed against the reference PBS group (G5) to determine changes in gene expression across different vaccination schemes. For the longitudinal comparisons each condition or sampling day was compared against the baseline reference, being day 1 when testing for expression changes after the priming dose or day 85 when testing for expression changes after the boosting dose. To determine expression differences related to the adjuvant usage at day 92 (boost), direct comparisons were performed between G1 (LAIV8/IIV5-AS03) and G2 (LAIV8/IIV5), and G1 and G5 (PBS). To determine genes with significant expression differences, Limma’s eBayes adjusted p values were corrected for multiple testing using the Benjamini-Hochberg (BH) method (*p* ≤ 0.05).

### Gene ontology enrichment analysis

Gene ontology (GO) biological process (BP), molecular function (MF), and/or cellular component (CC) enrichment analyses of differentially expressed genes were performed using the gProfileR R v0.6.8 package^49^. The background gene set was restricted to genes with detected expression in the filtered counts matrix. Genes ranked by log_2_ fold change were used as an ordered query. *P* values were corrected using the g:SCS algorithm to account for multiple comparisons.

### Gene set enrichment analysis

Gene set enrichment analysis (GSEA) was performed on a rank-ordered list of the longitudinal comparisons for each vaccine group. The ranking metric used was log_2_(FC) × –log_10_(*P* value). The analysis was performed with the fgsea package (v.1.18.0) for R (v4.1.0) with default parameters against the Hallmark database for GOBP (version 7.5.1) [PMID: 16199517].

### Cell-type gene signature enrichment analysis

Single-cell immune cell expression signatures derived from healthy PBMCs^16^ were used for gene set enrichment analysis against the DGE lists of each comparison to infer the cellular composition of the RNA-seq signatures. Enrichments were performed using Fisher’s exact tests and using Bonferroni correction for multiple comparison (*p* ≤ 0.05).

A second enrichment analysis was performed with the xCell method [PMID: 29141660] using xCell’s curated reference from ImmPort [PMID: 24791905] and filtered for blood cell types only (aDC, B-cells, Basophils, CD4 + memory T-cells, CD4 + naive T-cells, CD4 + T-cells, CD4 + Tcm, CD4 + Tem, CD8 + naive T-cells, CD8 + T-cells, CD8 + Tcm, CD8 + Tem, cDC, Class-switched memory B-cells, DC, Eosinophils, Erythrocytes, iDC, Macrophages, Macrophages M1, Macrophages M2, Memory B-cells, Monocytes, naive B-cells, Neutrophils, NK cells, NKT, pDC, Plasma cells, Platelets, pro B-cells, Tgd cells, Th1 cells, Th2 cells, and Tregs). The analysis was performed using xCell (v1.1.0) and the results visualized as a heatmap using heatmaply (v1.3.0) for R (v4.1.0).

### Reporting summary

Further information on research design is available in the Nature Research Reporting Summary linked to this article.

## Supplementary information

Supplementary Material

Supplementary Data

REPORTING SUMMARY

## Data Availability

Raw sequencing RNAseq data and relevant metadata has been deposited and is available at the NCBI Gene Expression Omnibus under accession number GSE217770. All processed data is available in the manuscript or the supplementary materials. The source data underlying Figs. 3A, B, 5E, F, and Supplementary Figs. 1 and 2 are provided as a Source Data file.

## References

[1] Erbelding EJ (2018). A universal influenza vaccine: the strategic plan for the national institute of allergy and infectious diseases. J. Infect. Dis..

[2] Krammer F (2018). Influenza. Nat. Rev. Dis. Prim..

[3] Perez-Romero P (2012). Reduced incidence of pneumonia in influenza-vaccinated solid organ transplant recipients with influenza disease. Clin. Microbiol. Infect..

[4] Arriola C (2017). Influenza vaccination modifies disease severity among community-dwelling adults hospitalized with influenza. Clin. Infect. Dis..

[5] Bernstein DI (2020). Immunogenicity of chimeric haemagglutinin-based, universal influenza virus vaccine candidates: interim results of a randomised, placebo-controlled, phase 1 clinical trial. Lancet Infect. Dis..

[6] Nachbagauer R (2021). A chimeric hemagglutinin-based universal influenza virus vaccine approach induces broad and long-lasting immunity in a randomized, placebo-controlled phase I trial. Nat. Med..

[7] Krammer F, Pica N, Hai R, Margine I, Palese P (2013). Chimeric hemagglutinin influenza virus vaccine constructs elicit broadly protective stalk-specific antibodies. J. Virol..

[8] Dreyfus C (2012). Highly conserved protective epitopes on influenza B viruses. Science.

[9] Kirkpatrick E, Qiu X, Wilson PC, Bahl J, Krammer F (2018). The influenza virus hemagglutinin head evolves faster than the stalk domain. Sci. Rep..

[10] Heaton NS, Sachs D, Chen CJ, Hai R, Palese P (2013). Genome-wide mutagenesis of influenza virus reveals unique plasticity of the hemagglutinin and NS1 proteins. Proc. Natl Acad. Sci. USA.

[11] Hai R (2012). Influenza viruses expressing chimeric hemagglutinins: globular head and stalk domains derived from different subtypes. J. Virol..

[12] Ng S (2019). Novel correlates of protection against pandemic H1N1 influenza A virus infection. Nat. Med..

[13] Aydillo T (2020). Pre-existing hemagglutinin stalk antibodies correlate with protection of lower respiratory symptoms in flu-infected transplant patients. Cell Rep. Med..

[14] DiLillo DJ, Tan GS, Palese P, Ravetch JV (2014). Broadly neutralizing hemagglutinin stalk-specific antibodies require FcgammaR interactions for protection against influenza virus in vivo. Nat. Med..

[15] Chromikova, V. et al. Activity of human serum antibodies in an influenza virus hemagglutinin stalk-based ADCC reporter assay correlates with activity in a CD107a degranulation assay. *Vaccine*, 10.1016/j.vaccine.2020.01.008 (2020). 10.1016/j.vaccine.2020.01.008 PMC7117648 31959425

[16] Hao Y (2021). Integrated analysis of multimodal single-cell data. Cell.

[17] Giudicelli V, Chaume D, Lefranc MP (2005). IMGT/GENE-DB: a comprehensive database for human and mouse immunoglobulin and T cell receptor genes. Nucleic Acids Res..

[18] Lee J (2019). Persistent antibody clonotypes dominate the serum response to influenza over multiple years and repeated vaccinations. Cell Host Microbe.

[19] Kerr MA (1990). The structure and function of human IgA. Biochem J..

[20] Bucasas KL (2011). Early patterns of gene expression correlate with the humoral immune response to influenza vaccination in humans. J. Infect. Dis..

[21] Tan Y (2014). Gene signatures related to B-cell proliferation predict influenza vaccine-induced antibody response. Eur. J. Immunol..

[22] Henn AD (2013). High-resolution temporal response patterns to influenza vaccine reveal a distinct human plasma cell gene signature. Sci. Rep..

[23] Hoek KL (2015). A cell-based systems biology assessment of human blood to monitor immune responses after influenza vaccination. PLoS ONE.

[24] Voigt EA (2018). Transcriptomic signatures of cellular and humoral immune responses in older adults after seasonal influenza vaccination identified by data-driven clustering. Sci. Rep..

[25] Guthmiller JJ (2022). Broadly neutralizing antibodies target a haemagglutinin anchor epitope. Nature.

[26] Blanchard-Rohner G, Pulickal AS, Jol-van der Zijde CM, Snape MD, Pollard AJ (2009). Appearance of peripheral blood plasma cells and memory B cells in a primary and secondary immune response in humans. Blood.

[27] Hammarlund E (2003). Duration of antiviral immunity after smallpox vaccination. Nat. Med.

[28] Kasturi SP (2011). Programming the magnitude and persistence of antibody responses with innate immunity. Nature.

[29] Nakaya HI (2015). Systems analysis of immunity to influenza vaccination across multiple years and in diverse populations reveals shared molecular signatures. Immunity.

[30] De Mot, L. et al. Transcriptional profiles of adjuvanted hepatitis B vaccines display variable interindividual homogeneity but a shared core signature. *Sci. Transl. Med.*10.1126/scitranslmed.aay8618 (2020). 10.1126/scitranslmed.aay8618 33177181

[31] Sciammas R (2006). Graded expression of interferon regulatory factor-4 coordinates isotype switching with plasma cell differentiation. Immunity.

[32] Ochiai K (2013). Transcriptional regulation of germinal center B and plasma cell fates by dynamical control of IRF4. Immunity.

[33] Kwon H (2009). Analysis of interleukin-21-induced Prdm1 gene regulation reveals functional cooperation of STAT3 and IRF4 transcription factors. Immunity.

[34] Reimold AM (2001). Plasma cell differentiation requires the transcription factor XBP-1. Nature.

[35] Nelms K, Keegan AD, Zamorano J, Ryan JJ, Paul WE (1999). The IL-4 receptor: signaling mechanisms and biologic functions. Annu Rev. Immunol..

[36] Shaffer AL (2004). XBP1, downstream of Blimp-1, expands the secretory apparatus and other organelles, and increases protein synthesis in plasma cell differentiation. Immunity.

[37] Hu CC, Dougan SK, McGehee AM, Love JC, Ploegh HL (2009). XBP-1 regulates signal transduction, transcription factors and bone marrow colonization in B cells. EMBO J..

[38] Li A, Song NJ, Riesenberg BP, Li Z (2019). The emerging roles of endoplasmic reticulum stress in balancing immunity and tolerance in health and diseases: mechanisms and opportunities. Front. Immunol..

[39] Tellier J (2016). Blimp-1 controls plasma cell function through the regulation of immunoglobulin secretion and the unfolded protein response. Nat. Immunol..

[40] Wrammert J (2011). Broadly cross-reactive antibodies dominate the human B cell response against 2009 pandemic H1N1 influenza virus infection. J. Exp. Med..

[41] Zost, S. J. et al. Canonical features of human antibodies recognizing the influenza hemagglutinin trimer interface. *J. Clin. Invest.*10.1172/JCI146791 (2021). 10.1172/JCI146791 PMC8321569 34156974

[42] Avnir Y (2016). IGHV1-69 polymorphism modulates anti-influenza antibody repertoires, correlates with IGHV utilization shifts and varies by ethnicity. Sci. Rep..

[43] Eisfeld AJ (2017). Multi-platform Omics Analysis of Human Ebola Virus Disease Pathogenesis. Cell Host Microbe.

[44] Livanos AE (2021). Intestinal host response to SARS-CoV-2 infection and COVID-19 outcomes in patients with gastrointestinal symptoms. Gastroenterology.

[45] Liao Y, Smyth GK, Shi W (2014). featureCounts: an efficient general purpose program for assigning sequence reads to genomic features. Bioinformatics.

[46] Li B, Dewey CN (2011). RSEM: accurate transcript quantification from RNA-Seq data with or without a reference genome. BMC Bioinforma..

[47] Law CW, Chen Y, Shi W, Smyth GK (2014). voom: Precision weights unlock linear model analysis tools for RNA-seq read counts. Genome Biol..

[48] Ritchie ME (2015). limma powers differential expression analyses for RNA-sequencing and microarray studies. Nucleic Acids Res..

[49] Reimand J, Kull M, Peterson H, Hansen J, Vilo J (2007). g:Profiler-a web-based toolset for functional profiling of gene lists from large-scale experiments. Nucleic Acids Res..

