# Supplementary material for: Transcriptome signatures preceding the induction of anti-stalk antibodies elicited after universal influenza vaccination
Source: NPJ Vaccines. 2022 Dec 10;7:160. doi: 10.1038/s41541-022-00583-w (PMC9741632; doi:10.1038/s41541-022-00583-w)
Supplement: Supplementary file 1 — Supplementary Material [file 41541_2022_583_MOESM1_ESM.pdf]

Supplementary Material

Title: Transcriptome signatures preceding the induction of anti-stalk antibodies after universal influenza virus vaccination

Authors: Teresa Aydillo[1,2*#]; Ana S. Gonzalez-Reiche[3*]; Daniel Stadlbauer[1]; Mary Anne Amper[4], Venugopalan D. Nair[4]; Chiara Mariottini[1]; Stuart C. Sealfon[4], Harm van Bakel[3]; Peter Palese[1,2,5]; Florian Krammer[1,6]; Adolfo García-Sastre[1,2,5,6,7, 8#].

Affiliations: *[1]Department of Microbiology, Icahn School of Medicine at Mount Sinai, New York, NY, USA; [2]Global Health and Emerging Pathogens Institute, Icahn School of Medicine at Mount Sinai, New York, NY, USA; [3]Department of Genetics and Genomics Sciences, Icahn School of Medicine at Mount Sinai, New York, NY, USA; [4]Department of Neurology, Icahn School of Medicine at Mount Sinai, New York, NY, USA; [5]Department of Medicine, Icahn School of Medicine at Mount Sinai, New York, NY, USA; [6]Department of Pathology, Molecular and Cell Based Medicine, Icahn School of Medicine at Mount Sinai, New York, NY, USA; [7]Department of Medicine, Division of Infectious Diseases, Icahn School of Medicine at Mount Sinai; New York, NY, USA; [8]The Tisch Cancer Institute, Icahn School of Medicine at Mount Sinai; New York, NY, USA*

** These authors contributed equally.*

[#]Corresponding authors. Email: adolfo.garcia-sastre@mssm.edu; teresa.aydillo-gomez@mssm.edu

Supplementary Table 1. Gene ontology categories enriched induced by adjuvant AS03 in the context of chimeric HA IIV5 vaccine.

Term Id	Domain	Term Name	Intersection	p-value
GO:0002250	BP	adaptive immune response	*TNFRSF17, JCHAIN, IRF4, IGLV1-44, IGLC2, IGLC3, IGHG1, IGHM, IGHV3-23, IGKV2-28, IGKV3-15, IGKV1D-39*	2.02E-07
GO:0006958	BP	complement activation, classical pathway	*IGLV1-44, IGHG1, IGHM, IGHV3-23, IGKV2-28, IGKV3-15, IGKV1D-39*	9.49E-07
GO:0030449	BP	regulation of complement activation	*IGLV1-44, IGHG1, IGHV3-23, IGKV2-28, IGKV3-15, IGKV1D-39*	1.40E-05
GO:0006909	BP	phagocytosis	*IGLV1-44, IGHG1, IGHM, IGHV3-23, TXNDC5, IGKV2-28, IGKV3-15, IGKV1D-39*	0.000154
GO:0034976	BP	response to endoplasmic reticulum stress	*ERLEC1, XBP1, TXNDC11, PDIA4, HSP90B1, TXNDC5*	0.00221
GO:0036500	BP	ATF6-mediated unfolded protein response	*XBP1, HSP90B1*	0.00944
GO:0050864	BP	regulation of B cell activation	*XBP1, MZB1, IGHG1, IGHM, IGHV3-23*	0.0157

GO:0031347	BP	regulation of defense response	*IRF4, HSP90B1, IGLV1-44, IGHG1, IGHV3-23, IGKV2-28, IGKV3-15, IGKV1D-39*	0.0364
GO:0072562	CC	blood microparticle	*JCHAIN, IGLC2, IGLC3, IGHG1, IGHM, IGHV3-23, IGKV3-15*	2.22E-06
GO:0042571	CC	immunoglobulin complex, circulating	*JCHAIN, IGHG1, IGHM, IGHV3-23*	0.00117
GO:0005788	CC	endoplasmic reticulum lumen	*PDIA4, HSP90B1, MZB1, TXNDC5*	0.00204
GO:0005576	CC	extracellular region	*JCHAIN, ITM2C, PDIA4, HSP90B1, MZB1, IGLV1-44, IGHG1, TXNDC5, IGKV3-15*	0.0398
GO:0003823	MF	antigen binding	*JCHAIN, IGLV1-44, IGLC2, IGLC3, IGHG1, IGHM, IGHV3-23, IGKV2-28, IGKV3-15, IGKV1D-39*	4.54E-10
GO:0008236	MF	serine-type peptidase activity	*SEC11C, IGLV1-44, IGHG1, IGHV3-23, IGKV2-28, IGKV3-15, IGKV1D-39*	1.69E-05
GO:0034987	MF	immunoglobulin receptor binding	*JCHAIN, IGHG1, IGHM, IGHV3-23*	0.00176
GO:0016864	MF	intramolecular oxidoreductase activity, transposing S-S bonds	*TXNDC11, PDIA4, TXNDC5*	0.00224

Supplementary Figure Legends

Supplementary Dataset 1. Longitudinal changes in gene expression and gene ontology (GO) enrichment analysis in G4 IIV8-AS03/IIV5-AS03 after prime.

Supplementary Dataset 2. Longitudinal changes in gene expression and gene ontology (GO) enrichment analysis in high versus low responders after receiving the IIV8-AS03/IIV5-AS03 (G4) after prime.

Supplementary Figure 1. Gene Set Enrichment Analysis (GSEA) of LAIV8 responses in G1 and G2.

Supplementary Figure 2. xCell enrichment analysis of blood cell type signatures for G4 on day 3 and day 7 after prime (A) and xCell enrichment analysis of blood cell type signatures for G1 on day 7 after boost (B).

Figure S1

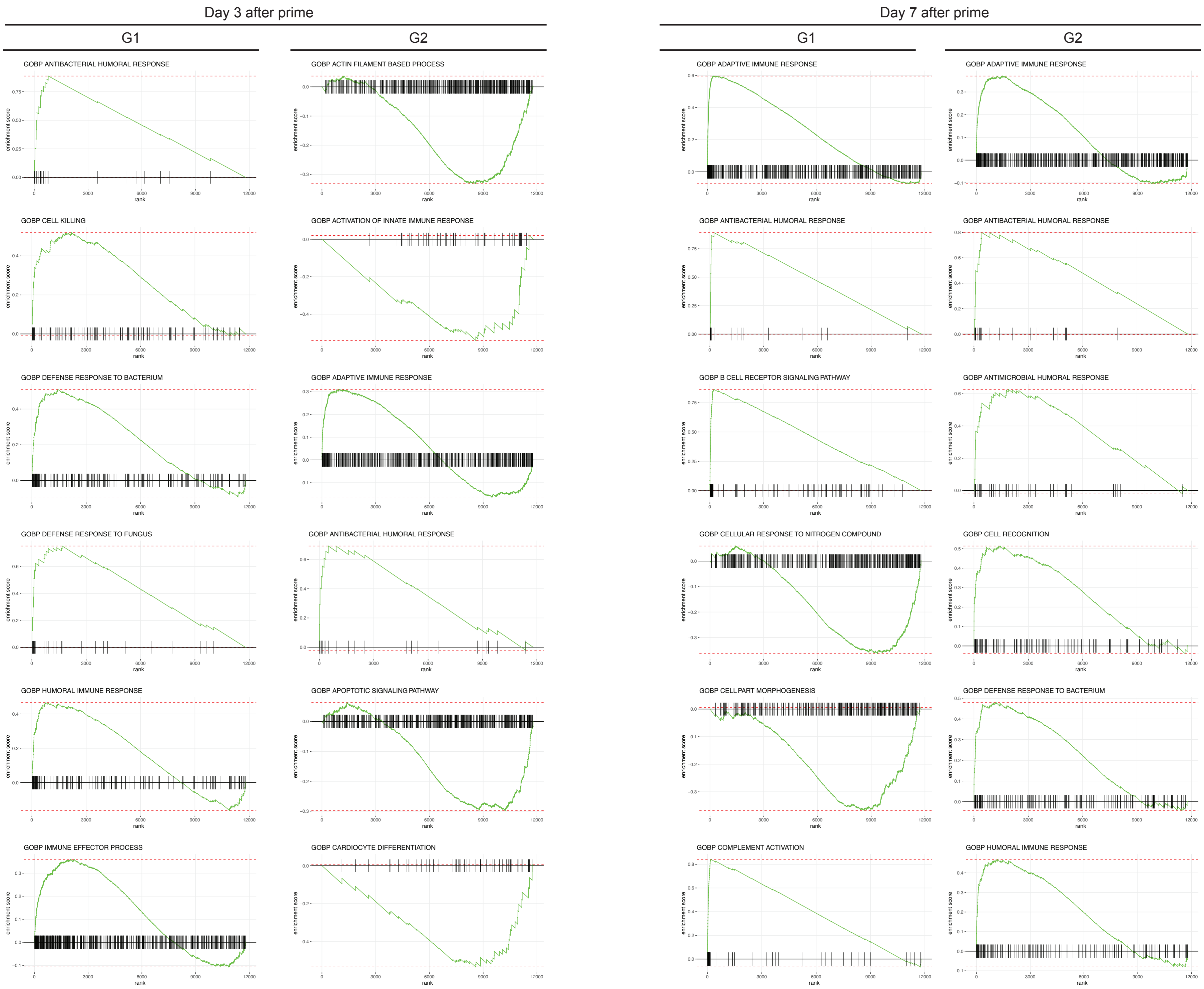
Day 3 after prime
Day 7 after prime
G1
G2
G1
G2
GOBP ANTIBACTERIAL HUMORAL RESPONSE
GOBP ACTIN FILAMENT BASED PROCESS
GOBP ADAPTIVE IMMUNE RESPONSE
GOBP ADAPTIVE IMMUNE RESPONSE
GOBP CELL KILLING
GOBP ACTIVATION OF INNATE IMMUNE RESPONSE
GOBP ANTIBACTERIAL HUMORAL RESPONSE
GOBP ANTIBACTERIAL HUMORAL RESPONSE
GOBP DEFENSE RESPONSE TO BACTERIUM
GOBP ADAPTIVE IMMUNE RESPONSE
GOBP B CELL RECEPTOR SIGNALING PATHWAY
GOBP ANTIMICROBIAL HUMORAL RESPONSE
GOBP DEFENSE RESPONSE TO FUNGUS
GOBP ANTIBACTERIAL HUMORAL RESPONSE
GOBP CELLULAR RESPONSE TO NITROGEN COMPOUND
GOBP CELL RECOGNITION
GOBP HUMORAL IMMUNE RESPONSE
GOBP APOPTOTIC SIGNALING PATHWAY
GOBP CELL PART MORPHOGENESIS
GOBP DEFENSE RESPONSE TO BACTERIUM
GOBP IMMUNE EFFECTOR PROCESS
GOBP CARDIOCYTE DIFFERENTIATION
GOBP COMPLEMENT ACTIVATION
GOBP HUMORAL IMMUNE RESPONSE
enrichment score
rank

Figure S2

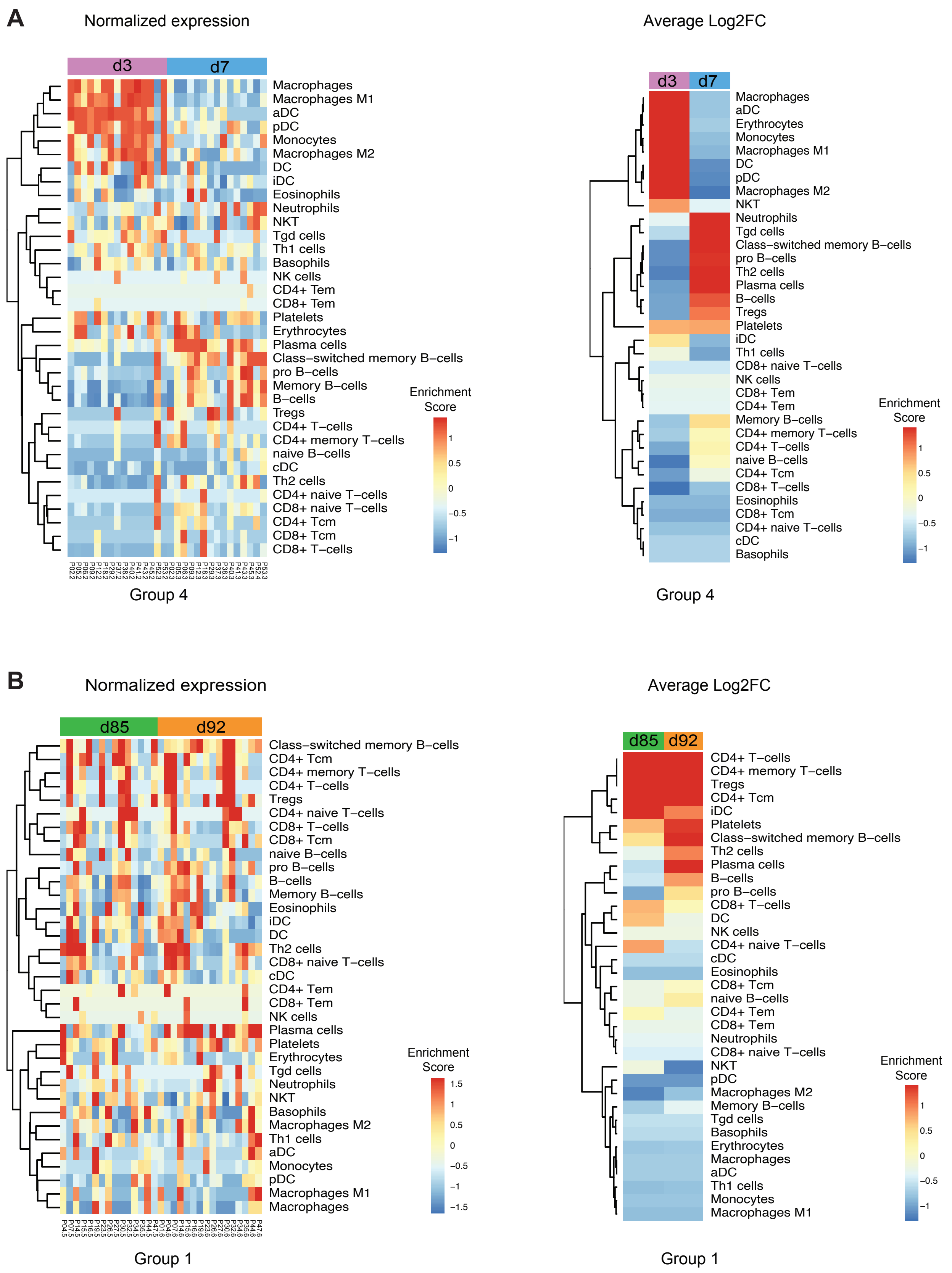
A
Normalized expression
d3
d7
Macrophages
Macrophages M1
aDC
pDC
Monocytes
Macrophages M2
DC
iDC
Eosinophils
Neutrophils
NKT
Tgd cells
Th1 cells
Basophils
NK cells
CD4+ Tem
CD8+ Tem
Platelets
Erythrocytes
Plasma cells
Class−switched memory B−cells
pro B−cells
Memory B−cells
B−cells
Tregs
CD4+ T−cells
CD4+ memory T−cells
naive B−cells
cDC
Th2 cells
CD4+ naive T−cells
CD8+ naive T−cells
CD4+ Tcm
CD8+ Tcm
CD8+ T−cells
Enrichment Score
1
0.5
0
−0.5
−1
Group 4
Average Log2FC
d3
d7
Macrophages
aDC
Erythrocytes
Monocytes
Macrophages M1
DC
pDC
Macrophages M2
NKT
Neutrophils
Tgd cells
Class−switched memory B−cells
pro B−cells
Th2 cells
Plasma cells
B−cells
Tregs
Platelets
iDC
Th1 cells
CD8+ naive T−cells
NK cells
CD8+ Tem
CD4+ Tem
Memory B−cells
CD4+ memory T−cells
CD4+ T−cells
naive B−cells
CD4+ Tcm
CD8+ T−cells
Eosinophils
CD8+ Tcm
CD4+ naive T−cells
cDC
Basophils
Enrichment Score
1
0.5
0
−0.5
−1
Group 4
B
Normalized expression
d85
d92
Class−switched memory B−cells
CD4+ Tcm
CD4+ memory T−cells
CD4+ T−cells
Tregs
CD4+ naive T−cells
CD8+ T−cells
CD8+ Tcm
naive B−cells
pro B−cells
B−cells
Memory B−cells
Eosinophils
iDC
DC
Th2 cells
CD8+ naive T−cells
cDC
CD4+ Tem
CD8+ Tem
NK cells
Plasma cells
Platelets
Erythrocytes
Tgd cells
Neutrophils
NKT
Basophils
Macrophages M2
Th1 cells
aDC
Monocytes
pDC
Macrophages M1
Macrophages
Enrichment Score
1.5
1
0.5
0
−0.5
−1
−1.5
Group 1
Average Log2FC
d85
d92
CD4+ T−cells
CD4+ memory T−cells
Tregs
CD4+ Tcm
iDC
Platelets
Class−switched memory B−cells
Th2 cells
Plasma cells
B−cells
pro B−cells
CD8+ T−cells
DC
NK cells
CD4+ naive T−cells
cDC
Eosinophils
CD8+ Tcm
naive B−cells
CD4+ Tem
CD8+ Tem
Neutrophils
CD8+ naive T−cells
NKT
pDC
Macrophages M2
Memory B−cells
Tgd cells
Basophils
Erythrocytes
Macrophages
aDC
Th1 cells
Monocytes
Macrophages M1
Enrichment Score
1
0.5
0
−0.5
−1
Group 1
